# Supplementary material for: Deletion of wfs1 Impairs Oligodendrocyte Precursor Cells Dorsal Distribution and Myelination Through the wfs1-hmgcs1 Axis in Zebrafish
Source: Biology (Basel). 2026 Aug 16;15(16):1402. doi: 10.3390/biology15161402 (PMC13510151; doi:10.3390/biology15161402)
Supplement: Supplementary file 1 [file biology-15-01402-s001.zip › File S1-Transcriptome sequencing data and differentially expressed genes (DEGs) in wfs1-- zebrafish at 5 dpf/2.KEGG/wfs1KOvsWT/UP/src/dre02010.html]

dre02010


- dre:571189

- Up regulated genes

571189(1.21484376268511)

- dre:570453

- Up regulated genes

570453\_1(4.66442924727155)
- dre:735310

- Up regulated genes

735310(1.16046701928099)

- dre:406803

- Up regulated genes

406803\_1(4.26869167669637) 406803\_2(1.03501206320206)

- dre:368620

- Up regulated genes

368620\_1(1.76749782511676)

- dre:570148

- Up regulated genes

570148\_1(1.99799176335957)

- dre:556826

- Up regulated genes

556826\_1(2.25777649220643)

Close
